# Navigating the Transition to Adulthood: Insights from Caregivers of Autistic Individuals

**DOI:** 10.1007/s10803-023-06196-z

**Published:** 2023-12-23

**Authors:** Samara M. Wolpe, Amanda R. Johnson, Sunny Kim

**Affiliations:** 1 Department of Education, University of California, Moore Hall, 457 Portola Plaza, Los Angeles, CA 90095 USA; 2 University of California, Santa Barbara, Professional and Continuing Education, Santa Barbara, CA USA

**Keywords:** Autism, Transition, Adulthood, Caregiver, Parent, Services, Qualitative analysis

## Abstract

With many teens having to transition from a mainly educational system of support to a set of health and social service systems (Shattuck et al. in Autism Res Treat 10.1155/2014/924182, 2017), there is a critical need to advance research and support services in the area of autism and transition to aid autistic* individuals and their families. This study aims to learn more about the experiences of caregivers of autistic young adults, their experiences navigating the transition process post-graduation, and what realistic steps could be undertaken by high schools, vocational schools, colleges, Regional Centers, and places of employment to ease this transition. Ten semi-structured interviews were conducted with caregivers of autistic young adults over the age of 18 focused on their experiences helping their children navigate the transition to adulthood. Using an iterative and inductive coding approach, three overarching themes were uncovered with twelve subthemes. The three major themes recurring in caregiver interviews were their experiences with navigating service receipt, exploring the landscape of opportunities available for their children, and the parent experiences specific to their role in their child’s transition into adulthood. Findings from this study provide a chance for stakeholders to learn from the lived experiences of caregivers navigating the frustration and confusion pertaining to transition for their autistic adult child due to the highly prohibitive access to service receipt, experiencing significant financial burdens, finding a niche for their children that fits their needs, desires, and talents, and managing their well-being.

The transition from adolescence to adulthood is a unique developmental phase for autistic teens and their families, marked by various obstacles. Autistic individuals share all the challenges of their neurotypical peers—pursuing higher education, searching for employment opportunities, navigating social relationships, and adapting to independent living. However, autistic individuals encounter additional hindrances to success that are often overlooked.

With many teens having to transition from a mainly educational system of support to a set of health and social service systems (Kuo et al., 2018), there is a critical need to advance research and support services in the area of autism and transition to aid autistic individuals and their families. A significant decrease in the availability of services and support systems characterizes the transition period for autistic youth. Parents and caregivers of autistic young adults have colloquially used the term “services cliff” to describe the sudden decline in service provision following high school graduation (Laxman et al., 2019). The consequences of the services cliff are far-reaching, negatively impacting the development and overall well-being of autistic individuals, as well as straining their interpersonal relationships.

Existing research has shed light on the pressing need for increased funding and support services designed for transition-aged autistic adults and their families. Several studies have documented the alarming decrease in service receipt post-high school, highlighting the challenges faced by this population. A national report conducted by the Drexel Autism Institute revealed a sharp decline in service utilization, with speech-language therapy rates plummeting from 66% at age 17 to a mere 10% after graduation (Roux et al., 2017). Moreover, over half of the autistic adults received no vocational or life skills services following high school.

Longitudinal studies have examined the changes in service provision over time, demonstrating that access to services dwindles significantly after graduation, with services becoming scarce even during the senior year of high school (Laxman et al., 2019). The scarcity of therapeutic services post-high school graduation, including speech, physical, and occupational therapy is particularly concerning, as these services are crucial for individuals' continued development and independence. Furthermore, there is evidence of inequitable distribution of services, with families of higher socioeconomic status receiving more support than those with lower incomes (Laxman et al., 2019).

There are limited opportunities for autistic adults in employment and higher education (Graetz, 2010; Roux et al., 2015a, 2015b). Employment rates for autistic adults remain very low, with a study by Roux et al., (2015a, 2015b) reporting that only 58% of autistic adults in their early 20 s are employed, a rate lower than individuals with other developmental disabilities, and even lower than those of ex-convicts. Of those with a job, autistic employees worked far fewer hours and earned lower wages than their typically developing counterparts (Cimera & Cowan, 2009). Autistic adults report difficulties in communication and social interactions with coworkers and employers, which is unsurprising given that many autistic individuals struggle with social skills, understanding of appropriate situational behaviors, and social communication skills (Cummins et al, 2020; Hendricks, 2010). Given these interpersonal challenges, many autistic adults have difficulties obtaining and maintaining competitive employment. Although these statistics are concerning for the outlook of autistic adults, there has been very little research that attempts to identify protective factors, interventions, and current frameworks in the legal and educational systems that promote this inequity, and how research can address this deficit.

Autistic adults also experience poor transition outcomes in post-secondary education, with 41% of autistic adults enrolling in a 4-year college (compared with 59% of neurotypical peers; White et al., 2016), and only 38.8% of those being able to graduate from college (Newman et al., 2011). However, there has been little research investigating the experiences of these individuals and their families that may have contributed to this high rate of attrition. As post-secondary education is a primary source of higher-paying competitive employment and increased odds of independent living (Ma et al., 2016), promoting access and accessibility within post-secondary education must be a priority of research conducted in transition-age autistic adults and their caregivers and families.

Parenting a child on the autism spectrum is associated with high levels of parental stress and depression as compared to parents of children with other developmental disabilities (Cohrs & Leslie, 2017; Hayes & Watson, 2013). This can be due to many causes, including the lack of support provided for families whose children need additional support. However, these mental strains have serious implications for the entire family unit, particularly for the autistic child, whose parents are generally their greatest advocates when navigating the school system, social relationships, and challenges they may encounter in the transition to adulthood (Boshoff et al., 2016; Stoner & Angell, 2006). Caregivers, particularly parents, are often the primary authorities on navigating the minutia of transitions from secondary school to post-secondary education or to employment for the transition-age autistic teenager or young adult. Therefore, understanding their perspectives, experiences, and recommendations for how this process can be improved and what steps can be taken to ease this process is a natural first step in the field’s attempt to understanding and improving transition outcomes for this population.

The present qualitative research study investigates the perspectives of caregivers of their autistic adult children in their experiences with service receipt and navigating periods of transition. The following research questions were investigated:

1. What were the experiences of caregivers of their autistic child who graduated high school in navigating the next steps of their lives?
2. What were some barriers caregivers encountered as they attempted to help their young autistic adult child succeed?
3. What resources did caregivers find most helpful in navigating this transition?

## Methods

### Participants

Ten caregivers of autistic adult children (ages 18 and over) were recruited for this study. Participants were enlisted either through an organization specializing in facilitating careers in the entertainment industry for autistic individuals or through personal networks. Convenience sampling was employed for participant recruitment, as the primary investigator utilized prior employment history with the organization.

Initial recruitment was conducted via email, where potential participants were invited to express their interest in the study. Those who indicated their interest were then provided with the consent form to review, along with a Google Survey to determine their eligibility by asking if participants were caregivers of an autistic young adult aged 18 or over. The survey also collected demographic information from eligible participants.

Participants were considered eligible if they were parents or caregivers of an autistic young adult over the age of 18 and lived in California. Residents of California were chosen due to the specificity of transition-related laws and the variation from state to state, rendering out-of-state transition-age experiences for families of autistic youth significantly different from the point of view of service receipt and policy.

All participants identified as women, and their autistic adult children’s genders were equally distributed between males and females. The autistic adult children’s ages ranged from 19 to 32 at the time of the interview. Demographic information for study participants, including their relationship to the autistic young adults, geographic location, gender, age, education, diagnosis status, ethnicity, and primary spoken language, is presented in Table 1. Participants were parents or caregivers of autistic young adults residing in California, with an even distribution of genders among their autistic adult children. The age range of the autistic adults varied from 19 to 32 years. Educational backgrounds included attendance at special education high schools (N = 5), mainstream schools (N = 1), a combination of mainstream and special education schooling (N = 3), and private schools accommodating both mainstream and special education students (N = 1). Nine out of the ten participants reported a formal diagnosis of autism for their child, while one participant reported a history of autism in addition to Down Syndrome. Ethnicity varied among the participants, with the majority identifying as White/Caucasian/European. English was the primary spoken language in all ten participants' homes.

### Materials

Materials for this study included a set of interview questions and the interview form. Questions and interview format can be found in Table 3. Google Surveys were used to establish eligibility, collect democratic information, and collect follow-up surveys about the participants' experience.

### Procedures

#### Development of Interview Protocol

The interview protocol was carefully developed to address the study’s specific objectives. The questions were designed to address the three aspects of the research questions: (1) the general experience of caregivers during the transition, (2) the difficulties they faced, and (3) the resources they found most helpful or would have utilized if they had been available. Furthermore, the questions were structured to emphasize participants’ suggestions for improvement to this transition process for future families and autistic young adults undergoing this transition. The semi-structured interviews consisted of 10 primary questions, with additional follow-up questions added as necessary to ensure a comprehensive exploration of the participants' experiences. The interview protocol was designed to allow for flexibility and adaptability during the conversations, ensuring that the flow of dialogue remained natural and participant centered.

#### Interviews

Interviews were conducted virtually through the Zoom platform allowing for face-to-face conversation with participants from the comfort of their home. The study team took calls from private rooms with headphones to ensure data security. These sessions were recorded using Zoom's recording feature to ensure accuracy in data capture. The Zoom-generated transcripts of the interviews were subsequently edited and anonymized by the research team. The Zoom-generated transcripts of the interviews were carefully reviewed and edited by the research team to ensure accuracy, as Zoom's automatic transcription feature did not consistently capture transcriptions correctly. The purpose of this editing process was to rectify any inaccuracies or omissions in the transcripts, thereby preserving the fidelity of the participants' responses and maintaining the integrity of the data analysis.

#### Participant Rights, Privacy, and Consent

Before recording began, participants were provided with the study's protocols and their rights as participants. Prior to filling out the eligibility form, participants were asked to review the consent form and ask any questions they had before the interview. Time was made during each interview before recording began to address any questions or concerns participants had about the interview, review the consent form if desired, and to explain the purpose of the study. They were also asked about their comfort level with being recorded, and consent was obtained before proceeding with the interview. Participants were given the right to withdraw their interview or edit their transcripts at any time. Additionally, member checking was utilized in the writing process before submission for publication to ensure participants felt their voices were accurately represented. Recordings and transcripts of the interviews were stored on a secure, password-protected online server. Access to this server was restricted to IRB approved research staff. This study was approved by the University of California, Los Angeles North Campus General Institutional Review Board.

#### Post-Interview Survey

After completing the interview, participants were sent a follow-up email expressing gratitude for their participation. They were also invited to complete an optional post-interview survey, providing an opportunity for them to share additional feedback on their interview experience and their overall level of satisfaction.

### Data Analysis

Data analysis followed the guidelines for thematic analysis proposed by Braun and Clarke (2006). An iterative and inductive approach was employed to code the data and identify recurring themes. The analysis involved multiple read-throughs of each transcript, during which the research team noted recurring sentiments and emerging ideas. The primary investigator then organized the identified themes into a codebook, a dynamic document that evolved throughout the coding process. Preliminary codes were created, and a synoptic chart was generated to capture the frequencies of each code's occurrence in the transcripts. Additionally, a tree diagram was constructed to illustrate the hierarchy of themes. The software Dedoose (Dedoose Version 7.0.23, 2016 ) was utilized to facilitate the analysis of the data. The ten anonymized transcripts were revisited by the primary investigator for a second round of coding, during which adjustments, refinements, and deletions were made to the codebook as needed.

Following the framework proposed by Willms et al. (1990), a systematic coding consensus approach was then employed. The two investigators independently coded transcripts. Subsequent meetings were held to discuss and reach a consensus on any discrepancies. This iterative process allowed the coders to collaboratively construct a robust coding system, fostering a shared understanding of the data and enhancing the credibility of the analysis.

#### Inter-Rater Reliability

To ensure the reliability and consistency of the coding process, a secondary researcher independently coded eight out of the ten transcripts. They coded the transcripts referring to the initial set of codes established by the primary investigator. Comparisons were made between the codes assigned by the secondary researcher and the primary investigator to assess fidelity.

## Results

The analysis of the 10 interviews revealed three significant themes: Service Receipt, Opportunities, and Parent Experiences. Coding and consensus procedures continued until the reliability coder and the master coder reached an agreement of over 80% (Syed & Nelson, 2015). This threshold was chosen to ensure a high level of consistency and reliability in the interpretation of the data. For frequency of codes applied to each major and subtheme, please refer to Table 2.

## Service Receipt

### Services Cliff

Nine out of the 10 parents mentioned experiencing some form of cessation of services after their children graduated from high school. One parent (Esme) lamented, “The day they turn 22, they are literally thrown in the street with nothing. There is nothing, there are no proper services, there is no housing services, there is no in-home services, there is nothing preparing them for the rest of their lives. So it's difficult.” Another parent (Olivia) expressed the sentiment that she felt as though “now that they’re adults, just nothing matters anymore.” The transition between high school to the next steps of life was compared frequently to the fear and uncertainty of receiving the child's diagnosis when they were first diagnosed with autism; the paralyzing doubt, both in a system which failed to provide them with clear guidance, and in themselves as decision makers in their children’s futures.

Many participants described the feeling of the rug being pulled out from under them, of trusted and helpful service providers being unable to help them, and of missing the safety and security they had built up from years of hunting down trusted employees, friends, and opportunities for their children that were only available to them before they reached adulthood or moved out of extended conservatorship (ending at age 22).

### Lack of Transparency

As caregivers begin what they describe as the arduous and opaque hunt to construct a new daily life full of opportunities and helpful services for their autistic adult child, many reported facing a lack of transparency on the part of service providers and government agencies in obtaining knowledge of what services they and their families are eligible to receive. One parent summed up the struggle by saying, “There are those programs, some of them exist. We just don't know where to find them, or how to find them” (Esme).

California Regional Centers are government-funded organizations that provide diagnosis and assessment of individuals with developmental disabilities and determine eligibility for services. They can help plan, access, coordinate and monitor the services and supports. Regional Centers (RCs) were a frequent topic amongst caregivers. While RCs were reported to be invaluable sources of information and gathering information about opportunities that their young adults might qualify to receive, in practice, accessing the information was fraught with difficulties. Parents reported either not receiving any information about the services, or receiving information about services in such a confusing manner that they were not able to parse out what inappropriate service might be. When asked what advice they would give to other caregivers who are making this transition, one participant advised that parents demand information from their local Regional Center: “A packet of all available services that that child is qualified to get… That'd be the first thing I’d say, because without that information, how are you supposed to figure it out?” (Olivia).

However, even with input from Regional Centers, caregivers still reported the experience of hunting down services and trying to determine a path forward for their autistic adult child was still prohibitively complex. One participant (Claire) lamented, “It's like going back to the drawing board at the very beginning, when you don't know what questions you're supposed to be asking for, and you don't know what services you're supposed to be asking for.” Even participants who believe they prepared themselves for the struggle ahead still reported an undue amount of confusion and effort in trying to determine a path forward, as captured by one participant:

“I didn't know what the heck was going on, and I considered myself pretty well- educated. I've been to law school. I've worked in special education, and I still was really confused about what was supposed to happen.” (Ella)

### Difficulty Accessing and Navigating Services

The difficulty of locating appropriate services for their child was a frequently expressed sentiment among participants. Many participants expressed the sentiment that, even when they found a seemingly suitable service that they thought would benefit their child, there was so much bureaucratic red tape that they were unable to obtain the service in time to use it and spent much of their free time fighting with service coordinators or attempting to get through to service professionals. One parent best summarized the experiences of wading through the restrictions put in place to limit access to services:

“It's a constant battle with Regional Center to get anything that you know benefits your kid. It’s so hard because they control everything, so you have to be polite… it's this constant churning of emotion because you want more for your kid and then you also understand why it's hard to get it, so there's this constant feeling like you're always in battle.” (Natalie)

Additionally, parents expressed frustration with navigating the Regional Center’s vendoring system. One participant stated:

“It’s so exhausting for the families, and then there's so much red tape… For example, they publish their list of vendors, but it's alphabetized, and for consumers of all age ranges for example, birth to 60 … well that's not helpful! I don’t need to know the name of the vendor. I need to know which vendors offer Adult Services, and what services they offer.” (Natalie)

Even those parents and caregivers who are able to get in touch with Regional Center coordinators and add themselves to the waitlist reported difficulty actually obtaining services. One parent (Liza) explained, “He's still living at home and we're in the process of trying to get him into supportive living, you know, we have an agency that agreed to work with us, but everybody's having a really hard time finding staff now so they're long waiting lists.” Even when services have been identified and the organization has agreed to provide the service, families still recalled waiting inordinate amounts of time to have the promised service come to fruition.

### Services Available but Unhelpful or Inappropriate

While many parents and caregivers struggle to obtain even basic services, participants reported that the services that are available are often unhelpful or inappropriate. Autism is a wide spectrum, with each individual having vastly different strengths and needs. As a result, participants expressed that finding appropriate services and post-graduation occupations can be a challenge. One parent explained:

“You always want to put your kid in a place where they’ll thrive and be challenged and be around people they can become friends with.” (Natalie)

One parent described the frustration she experienced when enrolling her son in what she believed to be a quality transition program, only to discover that the classroom was not only failing to provide him with adequate educational opportunities, but was actively unsupervised and unsafe for her son:

“So I went in… the classroom was so incredibly bad, and the aide was so bad… I just left, and I said to one of the aides I knew, please just keep him safe today. He came home that day, and he never went back…That was our transition. That was basically it.” (Ella)

Another participant reported similar troubles with transition and vocational programs, reporting that her son was engaged all day in activities he actively disliked: “When he finished high school…there were things like, learning how to garden, which he hates. Learning how to take care of animals, which he hates” (Esme).

Many participants reported being forced to pick between utilizing unhelpful, inappropriate, or even actively harmful services that did not promote their child's well-being, enhance their skill set, provide them with opportunities for growth, or going without services all together. Participants also described that most programs they were offered required the parents or caregivers to be present at all times, an option which is not feasible for many or most families who have other children and consistent employment. One participant described her experience of selecting from options as a dichotomy between being constantly present at her daughter’s side, or leaving her daughter at a program to be merely entertained and not occupied with meaningful work:

“A lot of the options that I see …you need to follow [your child]…A lot of those programs were more what I call autism babysitting, where they just want to have a kid in a program to keep them busy, or… give them pretty menial jobs that I didn't feel were very interesting, you know, to her… is that going to make her happy, and make her feel like she wants to go to work?” (Adele)

Another participant described her reaction when attending a resource fair at her child’s high school that advertised different ‘post-transition’ opportunities: “It was always like, oh, your child can maybe sweep or clean up a table, and I was like…I want more for [my son] than that, so there was really nothing offered” (Valerie).

Caregivers of autistic adults report similar struggles transitioning to higher education. One participant described the difficulty in finding a university that provides appropriate services for individuals with developmental disabilities, not just physical disabilities: “[My son] wanted to go to [college]. The Director of the learning center explained to me, we deal with physical disabilities, but everything else, they're on their own” (Esme).

Even basic services, such as reliable transportation, were reported to be a barrier to obtaining and reliably attending transitional programs or daily employment. One parent expressed, “Now we figured out the Access Van, we signed up with that, but from what I’ve been told by everybody is that it's not very reliable. So if you need to be at a class at a certain time it's not very reliable and that makes me nervous” (Valerie).

Participants explained that this chronic uncertainty creates an inability to plan one’s educational or vocational schedule reliably, leading to even further restrictions in opportunities for this population of emerging adults and increased burden on their caregivers to provide consistent transportation.

### Nonresponsiveness and Indifference

Nonresponsiveness and indifference on the part of service providers, college employees, and Regional Center coordinators is a common theme among participants' recollections. One participant indignantly recalled an incident where her son, who was enrolled in college in a musical performance class, was not permitted to perform at the final concert held for friends and family of the students because he missed a rehearsal. The reason he had missed that rehearsal was that the note taker who was supposed to write down the dates and times assignments, as her son has an executive functioning difficulty, was absent from several classes. Despite that this is a valid excuse, the teacher of the class barred her son from performing:

“[The teacher didn’t] let him perform because of this mistake [missing a rehearsal]? And all the teacher said was don't get me fired don't get me fired, which tells me she's thinking of herself…I wrote a letter to his… coach and said, you failed him… The teacher never got back to [my son], ever. And special services department never got back to me, ever. The message I’m getting loud and clear is that they don't care.” (Valerie)

Many participants reported similar interactions from various educational and vocational organizations, with one reporting that she was unable to get a response from her Regional Center until she called someone higher up in the organization, crying with frustration:

“I put in for the transportation from the Regional Center that same time and a year had gone by, and I still had no response from them... until I actually called my Regional Center worker’s supervisor and literally was crying hysterically to the supervisor saying, you people are supposed to help us, not hurt us, I’m going to have a breakdown because you're ignoring me, and it was so harsh. And they used the Covid excuse. Due to Covid we can't respond to an email.” (Olivia)

While some level of delay, wait lists, and bureaucracy is understandable from a large organization that is understaffed and underfunded, caregivers felt that wait times of a year for receiving important services are a failure of the support system during this already vulnerable time for autistic adults who need their services in a timely manner in order to be successful in this transition. Furthermore, participants reported that the indifference and nonresponsiveness displayed by these organizations were harmful to the mental health and well-being of families and young adults who are struggling through the systems to obtain necessary services. As one participant poignantly stated:

“When the Regional Center refused to process my transportation request and I literally reached out to her 25 times. That's when I felt like I wanted to quit… If they don't care, how am I supposed to do this?” (Olivia)

## Opportunities

### Abilities vs. Opportunities

Many participants reported struggles with finding services that fit their child’s abilities. Parents reported this difficulty from both sides of the spectrum, with some parents complaining that services required higher levels of executive functioning than their children were capable of, and other parents reporting that Regional Centers tended to gear their services more towards young adults who needed more support:

“If a family’s individual or young person has fewer or less challenges it can be easier to pick or get accepted into many different social, recreational, skill, or adult day programs. Their individual might be more comfortable and safe meeting at a Van pick up site, getting on public transportation independently or requiring less support, see (my daughter) can’t do any of that. There are few and far between programs that offer the specialized support she needs or their staff is not trained and paid well enough to provide the specialized support she might require." (Charlotte)

“In my experience, most of the Regional Center programs are geared towards those who need more support.” (Natalie)

The fact that parents on both sides of the spectrum are reporting similar if inverse difficulties suggests that it is possible there is an overall dearth of services serving either or both groups of individuals. It is a challenge to provide catch-all services for autistic individuals, as their needs are so varied and diverse. Participants suggested that parents or caregivers who are looking for services for their autistic adult child outside the realm of governmental or school support should first evaluate their child’s particular needs and abilities in order to exclude services that either require skills they do not have, or are geared towards individuals with more intensive and comprehensive needs.

Overall, most participants expressed a wish for more individualization in their services. As one participant put it, “There should be another program after high school like what I’m trying to create for her privately, that can really fill in the gaps, or that can bring…topic areas to her where they could be taught in a way that she can access them a little bit better… not necessarily a degree… It would just be, hey! I got to learn how to do this, and now I can work and do something I enjoy” (Adele). Many participants expressed a similar sentiment, that earning a degree was less important than the experience of learning, filling in the educational gaps from high school, learning independent living skills, and being amongst like-minded peers.

### Young Adult Level of Independence vs. Reliance

Young adulthood is often a time where individuals get to gain independence and differentiate from their family of origin (Schneider et al., 2016). Autistic adults, especially those who have extended conservatorship (whose caregivers, often parents, continue to have decision making power over their daily living, medical, and legal decisions, similar to when they were under the age of 18), often struggle with balancing increasing independence and relying on caregivers for necessary assistance (Cribb et al., 2019). As one parent stated, “I think a big part of this process, for me…was trying to recognize his desire to be independent and his frustration at not being able to be independent” (Liza).

Parents of autistic adults who are able to take increased ownership in independence over their everyday lives reported excitement, with one participant saying, “she's now participating in her life in a way that she definitely was not before, so I can let go, I can pull back now, I feel like we are crossing some kind of finish line, and I’m so excited” (Olivia). This participant also recalled an instance where her daughter left the house for a day, and despite her fear, she allowed her to go and have a formative experience:

“I have secretly raised the bar for [my daughter] all the time….She's getting ice cream with these typical girls, something bad’s going to happen, but I let her go despite my fear… I love the fact that my daughter was in the world, having a good time without me. That was the best gift I could have.” (Olivia)

Some parents participating in the study reported the opposite experience of their autistic adult child unable to partake in many independent experiences on their own. One participant remarked on this disparity, saying, “Different individuals… say I can’t wait to move out and get my own apartment, [but] that’s not [my daughter]” (Charlotte).

It is the goal of most caregivers of individuals in this age group to promote as much independence as possible. Participants discussed the difficulty of looking for residential programs and other services to help their young adults live out of the house independently, with one participant explaining that even assisted living would be difficult to manage for her son: “Residential programs… there is one that is starting [to be] built … it’s for very high functioning kids. …I could never do that with [my son]” (Esme).

Parents also reported that, with increased service needs, finding residential programs that suit their child is even more difficult. In many cases, parents reported fear and anxiety about leaving their children alone. One parent described why finding a residential program that is appropriate for her daughter is nearly impossible:

“[My daughter] has to have a lot of support, because she pretty much has to have 24/7 support… I’m here…because someone legally has to be here, she can’t be left alone in the house, it's not safe.” (Charlotte)

However, levels of independence of which the young adults desire and are capable varies greatly. For autistic adults who attend college, the transition from reliance to independence can be nearly total, as colleges do not allow parents of their students to check up on their progress, grades, or communicate with their professors or career counselors. While this policy is logical for students who value their privacy, many autistic adults who have executive functioning difficulties need accountability of a parent, guardian, or caregiver of some kind to ensure that they are not slipping off the radar. One parent described the frustration she felt her son experience after going to college, but then failing out of school:

“In college I just remember feeling at a loss…nobody would talk to us directly because he was 18…so I just felt like I kind of had to throw my hands up in the air and be like, okay, I guess you're going to…handle this.” (Helen)

While it is critical for autistic adults to obtain their freedom and independence, it is also important for these individuals to receive the support they need to be successful at their academic and vocational endeavors. This balancing act is one that arose repeatedly as participants described their struggles in supporting their adult children, and one in which they are provided with little to no guidance or support.

### The “Right Fit"

Every participant in the study expressed the desire to find an occupation or educational experience for their autistic adult child that was fulfilling and felt like the “right fit" for their interests and desires for the future. Dedication to passion and special interests are often hallmarks of autistic young adults, coupled with a decrease in tolerance for enduring activities that are not of interest to them (Jordan & Caldwell-Harris, 2012). However, participants reported that most vocational and job training programs they encountered are geared towards the students gaining experience in many different professions, with young adults participating in the program being required to rotate through several mandatory service positions regardless of personal interest or talent. One participant described her dissatisfaction with the way jobs were assigned, commenting:

“[They should] probably also have much more clear paths for people….they have a job at the local pet store here…that's your first job, or whatever. But I also feel like it should be a little bit based on the person's interests. Not everyone should have to just go work in a pet store.” (Ella)

While many participants expressed that they felt that college was either suggested to them as the ideal next step for promoting and ensuring their child's future by their high school, service providers, or society in general, few participants reported positive experiences for their children in college. One participant expressed her frustration with trying to help her son navigate the college system that did not provide him with appropriate accommodations, saying, “I feel like all we’re trying to do is trying to fit a square peg into a round hole this whole time” (Valerie). Several participants reported deciding to postpone college or not to attend entirely in favor of activities they felt would be a better fit for their children. One participant described her conviction that her daughter was better off focusing on post-high school internships and narrowing down her interests before pursuing any higher education:

“We felt like any post high school learning would be too abstract for her. We thought she really would benefit more from first getting some real-world experience in her career interests first, and then perhaps going to school if it made sense later on.” (Natalie)

All participants reported that it was of great importance to them that their autistic adult child find an educational or vocational situation in which they felt accepted, interested, and valued. When asked what advice they would give to other parents or caregivers who navigating the transitional period for their child, one participant responded:

“I think I would maybe have [parents of other transition-age autistic youth] kind of spend a little time thinking about….what they feel would help their child… access the world, and communicate in the world better and more fluidly. Like what would make their child happy… I want [my daughter] to continually be learning and growing… I don't want her to just do something that's super boring just because she can do it.” (Adele)

Overall, participants reported that it was extremely important to them to make sure that their children were continuing to learn and grow, pursue their interest, and find a place for themselves in the adult world where they could continue to develop, both personally and professionally.

## Parent Experiences

### Anxiety About the Future/Finances

Participants expressed anxiety about a myriad of concerns about the future of their children, their families, and the financial strain placed upon them by the need to continually support their adult children, who may not have an income of their own.

Financial strain was an almost ubiquitous concern amongst the participating caregivers. The cost of taking care of an adult with limited earning potential and continued dependence on their parents was felt to be substantial amongst the caregivers. One participant described the stress of planning financially for their child's future:

“Is she going to be able to make enough money to provide for herself? Well probably not, and there’s still a big question mark about what her abilities will be. And that of course makes it a little hard to plan…we’re not just saving for our retirement, we're saving for another entire person's retirement. And it's not just a retirement, it's from age 22 on…. we don't really know if she's going to be able to contribute any money to her bank account.” (Natalie)

In addition to saving for the future, participants contended with the financial difficulties surrounding hiring and obtaining quality service providers. While Regional Centers and some government organizations sponsor salaries for certain vendored service providers, parents reported that the compensation can be low or minimum wage for extremely taxing work. One participant described the difficulty of finding quality service providers on such low wages:

“The hourly rate is very, very low…Who are you finding to work for $16 an hour? Not very many people, not very many very qualified people. So, you sometimes have to get a little bit resourceful…caregiving and people who work with individuals with disabilities are severely underpaid, but they're also not trained to the level that they should be trained so when you find somebody who has a deep interest in working in the field, they need to be honored, they need to be properly compensated… it's a very difficult field for people to stay with, but I think it's because they're not being paid enough.” (Ella)

Despite low wages and the high cost of living for certain Regional Center funded service providers, families are prohibited from legally supplementing those wages. However, caregivers reported are at times being forced to surreptitiously pay service providers supplementary wages to keep quality service providers, assuming they are financially able to do so. Participants expressed that paying service providers under the table places an additional undue burden on already financially strained families just to keep the status quo of necessary and essential services.

“So I think for people like me, and you know and others I know it's, you know, everybody works outside of the system, you know, even if you're with an agency and finding your own people, you have to, you know, pay them under the table, or they won't stay.” (Claire)

In addition to these monetary strains, nearly every participant expressed fear of what was going to happen to their child after they are gone, both financially and in terms of caretaking for their needs. One participant summarized the shared sentiments of the fear of the future of their autistic adult child:

“The hardest part for parents is that you know we're going to be gone. There'll be a point where we're gone and…And I’m going to be completely blunt here, and I can tell you there are a lot of parents who say, I only wish that they're gone before I am so that I don't have to worry that someone's going to take care of them as well as I can.” (Charlotte)

Participants report constantly balancing the fear of the future and the need to take action to prepare for the child's eventual independence when they are no longer around to take care of them, but at the same time, the need to act calmly and from a place of information and education rather than fear.

“I think that the first thing to do is to prepare the parents. Talk to the parents. Let the families know what is out there, what you can expect, what you cannot expect, what you can ask for, and how are you going to pay for this? How are you going to manage this in your life?” (Esme)

### Other Parents as Resources

When asked where participants obtained the resources they currently use for themselves and their children, most caregivers cited other parents and caregivers in the community as the dispensers of vital information. The organizations such as Regional Centers, colleges, and other service providers were seen as too heavily laden with bureaucracy and confusing red tape to provide helpful information, and other parents were viewed as trusted veterans of an untrustworthy system.

Parents who had already navigated through multiple service providers were believed to know the best of the available options. Participants recommended to parents and caregivers new to this process to “arm yourself with advocates and professionals who… are familiar with this landscape. I think that would definitely help, because there are so many services that they don’t tell you about that are out there. The only way to find out is if you talk to other parents” (Natalie).

The types of information conveyed from parent-to-parent spans from services to programs, to shortcuts to cut through some of the bureaucracy and red tape surrounding obtaining information and necessary services. One parent recalled that when her daughter was first diagnosed as autistic when she was only a few years old, it was the community of parents who guided her through the process rather than medical professionals:

“[The doctor] would say, yeah, I think the child has autism, I’m giving you an autism diagnosis, and then they just send you on your way out into the wilderness… you really don’t have any guidance and you don’t know what to do with that or where to turn. Luckily I was led to parents, other parents, so right away, I was put on a pathway that I’m so grateful that we were put on but I needed somebody to tell me, okay, you need to call the Regional Center. And calling the Regional Center wasn’t enough, you had to call the Regional Center and say, my child is banging their head against the wall.” (Claire)

Caregivers repeatedly reported a deep sense of trust built into the community of autism caretakers that surpassed the eroded trust in the establishments that were supposed to assist them. The community’s benefits went beyond information and into self-protection. When one participant recounted a confrontation she had with a professor who mistreated her son and was discriminatory against him due to his executive functioning difficulties, she described how another ‘autism mom’ went to bat for her against the school: “She… confronted her and said…the autism community of moms and parents is very tight knit, and we hold each other” (Valerie).

### Parental Burden

“It just ends. Everything ends. High school and all that support ends. And then you have to really figure out, what do you want to do with your kid? And what do you think is best for your kid? And then you have to do all the work yourself to research what’s out there, and what programs are out there… They basically literally sent me an email with links on it. [laughs]…It’s a lot of work figuring it out.” (Adele)

Although nearly every caregiver expressed their deep affection and dedication to their adult children, they also reported that a huge burden came along with navigating the system set up to transition them into adulthood. High school ends, and the burden shifts almost entirely onto the caregivers to ensure their young adults’ success. Parents and caregivers report not being given many guideposts. As the participant above recalls, many parents are simply sent an email with links to potential resources for their children, with no other transition materials. However, participants repeatedly emphasized the importance of differentiating the stress and heartache of dealing with these systems from how much they love their child and are willing to be there to support them: “The hardest part is usually, it's not the autism and it's not [my daughter], it's the servicing, it's the industry. It's the paperwork of autism” (Olivia). Many participants reported the sentiment that it was not their children themselves that made the transition to adulthood difficult, but the systems put in place supposedly to help them succeed.

Importantly, several caregivers expressed frustration at the intersectionality between being a single parent and dealing with the paperwork of autism. One participant pointed out:

“I’m a single parent and…we have it harder than couples…Here in a couple everything's easier, you get a tax break …you get everything...I’m also working full time… It's just me and so why isn't that delineated, you know?” (Olivia)

Overall, participants expressed a deep exhaustion at the need to be continually involved to the extent that they were in their children's lives. One participant said, “Kids… it's like, at this time in their lives they go off to college, there's a reason, there's a biological reason. [laughs] Because I’m tired. I’m so tired. [laughs] You know, like, ugh!!!” (Claire). Another participant expressed that the workload of navigating these systems had interfered with her career: “I don't get to do enough of my own work…I’ve given up my career because of this” (Charlotte).

### Positive Educational, Work, and Service Experiences

Most positive experiences participants relayed in the interviews were related to service experiences with service providers who truly cared and understood the autistic adult. Some of these service providers were not even specialists in autism–several participants recollected stories of higher education professors and staff that connected with these adults and were willing and open to working with their challenges and embracing their talents. One participant recalled a professor who continued to work with and support her son who dropped out of college due to the demands becoming too overwhelming:

“He does have a good relationship with one Professor… [The professor] responded really well to him when he said he wanted a leave of absence…he said, you do what you need to do. And he's been there for him, and so I do think that relationship without his relationship with that one Professor has made a difference in terms of him feeling like, oh, somebody… would appreciate me being back there, besides my parents.” (Helen)

Participants expressed intense gratitude and appreciation for positive experiences with service providers, with such comments as: “I feel like I’ve had great service providers… the current one is unbelievable. I mean, I’d give that woman my house, [name of therapist] who runs that therapy because it's so great” (Olivia). Due, perhaps, to the intense frustration and immense amounts of labors on the part of caregivers to obtain services, useful services and compassionate, effective service providers were subject to the greatest praise and considered irreplaceable.

Although positive service providers and educators made huge improvements in the lives of autistic adults and their families, some participants described valuable lessons and positive experiences gleaned even from classes that were not a perfect fit for their young adult’s interests:

“He took a class in customer service and of course he's never going to do customer service, he's not verbal. But he said it was actually the best social skills class he's ever taken…he said, ‘I learned how to deal with assholes.’” (Liza)

Overall, participants reported that the most valuable service, work, and educational experiences were those where the educators or service providers or employers were kind, compassionate, open to learning about the individual, and willing to work within the system to create accommodations so that the young adult on the spectrum was set up for success.

## Discussion

This study utilized the principles of community-partnered research through conversations with caregivers and parents of autistic adults. Many participants expressed gratitude that someone in the research community was willing to listen to their voices, as they felt unheard by the research community, their service providers, and the world.

The importance of addressing the barriers to service receipt for this population is evident in the participants’ lived experiences. Experiences such as forcing parents and caregivers to comb through long lists of alphabetized vendor names, each of which must be researched to find out what their purpose is and what age group they serve, or waiting up to a year to receive necessary services, highlights the precarious position many families in this community find themselves navigating post-high school graduation. Changes such as improving the response rate on the part of Regional Centers and other organizations that provide services, simplifying the information and avenues of service receipt, and increasing communication between families and service providers may be essential steps in lessening the stress in caregivers and improving success rates in autistic adults who are pursuing opportunities in adulthood that are appropriate to their interests, abilities, and talents.

While it is not necessarily a waste of time for young adults to have to work in jobs that they find less than entirely satisfying or aligned with their interests as it may teach endurance, patience, and perseverance in the face of professional endeavors that are not to one’s preference, it does not adequately prepare them for future vocations they may pursue out of school. Learning more about the opportunities that autistic adults receive and the barriers to success in college and employment from a logistical perspective is likewise critical in understanding the poor success rates in adulthood in this community. Lastly, understanding more about parent and caregiver experiences in navigating this transition can help elucidate the areas of support that need to be strengthened in educating and empowering families, and lessening the burden on families and autistic individuals.

This study has several notable limitations. Although each interview was lengthy and rich in content, the experiences of 10 individuals cannot be generalizable to all caregivers and parents of autistic adult children. Furthermore, participants in the study are majority white, meaning that their experiences are partly shaped by those characteristics. Finally, while the participants in this study are close to the autistic adults, they cannot speak for them. An important follow-up to the current study would be to conduct a similar series of interviews with autistic adults to investigate their experiences in transitioning to adulthood.

In conclusion, this research adds to the literature by highlighting the dire need for better transitional programs and services for autistic adults. Additionally, the findings from this study provides a chance for stakeholders to learn from the lived experiences of parents navigating the frustration and confusion pertaining to transition for their autistic adult child due to the highly prohibitive access to service receipt, experiencing significant financial burdens, finding a niche for their children that fits their needs, desires, and talents, and managing their well-being. Lastly, the impact of positive experiences with service providers, educators, and work experiences should not be underestimated. The autistic community has a great deal to give to the world through their unique talents, interests, and abilities. Facilitating a smoother transition into adulthood and removing some of the burden from families during this time of transformation is an important step in creating a world in which autistic youth can become successful and fulfilled adults.

## Appendix

See Tables 1, 2 and 3.

**Table 1:** Demographic characteristics of caregiver and adult child

Characteristics	n	%
Caregiver gender		
Male	0	0
Female	10	100
Child gender		
Male	5	50
Female	5	50
Child diagnosis		
Autism	9	90
Down syndrome and history of autism	1	10
Child racial/ethnic background		
White	8	80
Italian	1	10
Hispanic	1	10
Type of high school		
Private	1	10
Special education (SpEd)	5	40
Mainstream	1	10
Combination (mainstream/SpEd)	3	30
Child’s age	Range: 19–32; *M* = 23.6 (*SD* = 3.8)

**Table 2:** Code frequency and application

Code name (major themes bolded)	Participant number and pseudonym										
	P_10 'Ella'	P_09 'Adele'	P_08 'Esme'	P_07 'Charlotte'	P_06 'Olivia'	P_05 'Helen'	P_04 'Eliza'	P_03 'Claire'	P_02 'Valerie'	P_01 'Natalie'	Totals
**Opportunities**	**8**	**17**	**6**	**6**	**4**	**10**	**7**	**1**	**15**	**5**	**79**
Abilities vs. opportunities	0	5	2	2	0	0	1	0	3	2	15
Young adult level of independence vs. reliance	0	1	1	3	3	8	4	0	5	0	25
The “right fit”	4	14	3	2	1	2	5	1	10	2	44
**Parent experiences**	**19**	**16**	**20**	**27**	**26**	**10**	**11**	**18**	**18**	**11**	**176**
Anxiety about the future	0	0	4	8	4	1	0	4	2	2	25
Finances	7	3	5	3	1	1	1	2	9	2	34
Other parents as resources	2	0	2	0	1	0	0	4	2	1	12
Parental burden	2	5	7	10	6	1	1	2	9	2	45
**Service receipt**	**23**	**20**	**25**	**23**	**31**	**23**	**17**	**20**	**36**	**20**	**238**
Difficulty navigating/accessing services	1	2	2	1	9	1	4	3	4	8	35
Lack of transparency	6	0	4	0	3	7	0	7	4	2	33
Nonresponsiveness/indifference	0	0	1	1	8	1	1	2	9	3	26
Positive service experiences	5	2	3	5	5	4	1	1	7	1	34
Services available but unhelpful	3	7	8	2	2	0	3	3	5	3	36
Services cliff	6	5	6	4	4	0	2	5	1	4	37

**Table 3:** Interview protocol

Interview protocol
*Beginning of transition planning process*
When did the transition planning process begin for your child/ren when they were in high school?
What did the services consist of? What did the process look like?
What was the transition process like for you as a parent navigating this?
*Transitioning post-high school*
What did the services look like for your child/ren after they graduated high school?
How has COVID-19 impacted service receipt or the transition process more generally?
*Overall*
What do you think every family should know about the transition planning process?
What would you tell someone who is just starting in the transition process to prepare them for the “services cliff”?
What could schools or policy makers could do to make this process easier for you and your family?
If you oversaw policy around the importance of transition planning, noting the multilevel needs in and complexity of the transition process, what would you do?
How do you, as a caregiver, take care of yourself as you navigate these challenges?
